# The Promissory Visions of DIYbio: Reimaging Science from the Fringe

**DOI:** 10.1080/09505431.2022.2028135

**Published:** 2022-01-17

**Authors:** Sonja Erikainen

**Keywords:** DIYbio, fringe science, promissory visions, sociology of expectations

## Abstract

Recent years have seen a proliferation of do-it-yourself biology (DIYbio) initiatives, consisting of people undertaking a range of bioscience activities outside traditional research environments. DIYbio initiatives, while diverse, exist at the fringes of institutionalised science, which enables them to advance different promissory visions about what science, especially bioscience, could or should become in the future, including how it should be governed. These visions reconfigure conventional delineations of science in politically and normatively loaded ways that can simultaneously reaffirm, contest, and shift the traditional epistemic foundations of science. They put forth alternative science futures in ways that highlight the performative force of promissory visions in shaping not only mainstream but also fringe science activity. DIYbio offers a fruitful lens for understanding how science is currently being reconfigured by unconventional actors to encompass new meanings and domains. It offers a different angle on the wider sociology of expectations engagement with the future as an analytical object, by showing how the future of science is constructed and managed from the fringe. Yet, DIYbio initiatives' promissory visions are also embedded within neoliberal ideals of productive and entrepreneurial citizens, highlighting how the wider socio-economic context constrains the alternative futures manufactured by these initiatives.

In recent decades, there has been growing interest and investment in public engagement with science, and professional scientific institutions and research funding bodies have been progressively promoting public involvement as a key part of scientific research. While citizens and publics and so-called lay people have conventionally been positioned as recipients of knowledge and services provided by science professionals, scientific institutions and funding bodies now advocate multilateral engagement, involvement and participation approaches between scientists and citizens (Lewenstein, 2003). Concurrently, national and international governance systems are increasingly attempting to reimagine the relationship between citizens and the state, moving towards participatory citizenship models that involve citizens as co-producers and partners in public service design and delivery (Newman, 2001). Governments have also been promoting neoliberal discourses encouraging citizens to be active and entrepreneurial contributors, taking responsibility for their own lives rather than relying on government actors to provide and govern for them (Bloom and Kilgore, 2003; Drake, 2011).

In this context, initiatives of various sizes and shapes consisting of people undertaking bioscience activities outside traditional research environments, like professional science institutions and laboratories, have proliferated. These initiatives have multiple names e.g. citizen biology, biohacking, and do-it-yourself biology (DIYbio). People involved in them generally organise in digital spaces and community laboratories (Meyer and Vergnaud, 2020) that provide equipment (often crowd-sourced or self-assembled), materials, and protocols for members to do bioscience. Activities include DNA barcoding and genome sequencing, tinkering with yeasts and other microbes, monitoring the environment, and undertaking biomedical experiments (see e.g. Grushkin, Kuiken, & Millet, 2013; Seyfried *et al*., 2014). DIYbio exists at the boundary edges of institutionalised and legitimate(d) science, forming a liminal sphere of activities that take place at its ‘fringes’ (Vaage, 2016). As such, it epitomises a form of science-making where people outside traditional research environments are forcefully active in practicing science and producing knowledge, in ways exceeding mere public ‘engagement’ and ‘involvement’ in scientific activities conducted within professional institutions and laboratories (Kelty, 2010).

This paper takes DIYbio as a lens to explore how science is currently being re-delineated and re-negotiated by unconventional fringe actors. It asks: how do DIYbio initiatives conceptualise science, and what political or normative perspectives and contextual developments underlie these conceptualisations? The paper draws on qualitative thematic analysis of DIYbio initiatives’ websites, focusing on how DIYbio initiatives represent themselves through their aim, mission, value, and normative statements. It builds on the sociology of expectations to argue that DIYbio initiatives construct future-oriented promissory visions about what science could or should become. These visions direct DIYbio activities and work to (re)configure science, including how it should be governed, in politically and normatively loaded ways that simultaneously reaffirm, contest, and shift the traditional epistemic foundations of science. While DIYbio initiatives are diverse, their location at the fringes of institutionalised science means that they are generally not strictly bound by professional and institutional scientific conduct norms and standards, enabling them to imagine and practice science differently. They foster alternative promissory futures that scholars should understand in the context of broader socio-economic developments, including the increasing promotion of neoliberal and entrepreneurial citizenship discourses.

Promissory visions around DIYbio can offer a different angle on the wider sociology of expectations literature by showing how people outside professional scientific institutions manufacture alternative futures at the fringes of institutionally legitimated science. These futures involve new actors and heterogenous science activities that intentionally move beyond the conventional boundaries of science, highlighting promissory visions’ performative forces in shaping fringe and mainstream science activity. They also highlight the importance of broadening STS scholars’ engagement with the future as an analytical object by looking at how the future is constructed and managed not only by conventional actors at the centre of scientific innovation but also by new unconventional actors located at the margins. In so doing, this paper sheds new light on the shifting and plural meanings of science in the present context in which citizens and publics are increasingly positioned as active contributors to science, but in ways conditioned by governments’ overarching drive to producing active, entrepreneurial, and self-governing citizens.

## Analytical Perspectives: Fringe Science and Promissory Visions in the Context of Neoliberal Discourses

DIYbio is a part of broader contemporary DIY activity, in which people are engaged in a diverse variety of do-it-yourself practices, from making electronics to producing their own food (see Davies, 2017). DIY activities can collectively be understood to embody a challenge against professionalisation and exclusive notions of expertise in various forms, representing ‘a shift away from relying on experts and professionals towards self-reliance and independence from the larger systems of governance and capitalism’ (Wehr, 2012, p. 57). The DIY movement is generally based on the idea that ‘the tasks that many are ready and willing to have others do for them can (and perhaps should) be done by one’s self,’ and DIY activities have been positioned as a solution to experiences of loss of control in relation to structural social forces that may felt to be beyond individuals’ influence (Wehr, 2012, p. xi). Ratto and Boler (2014), among others, have argued that DIY activities can be understood as a key component of emerging modes of ‘DIY citizenship,’ encapsulating the politically interventionist ways in which citizens are developing distinctly DIY products and processes without relying on the state to provide for them.

DIYbio represents a challenge to the idea that science is under the exclusive purview of professional scientists or conventional experts undertaking research and developing technologies in university and industry laboratories accessible only to those with appropriate credentials and professional status. It is one form of subverting the sphere of (legitimated) science’s exclusion of outsiders and alternative kinds of expertise. This sphere has conventionally suppressed epistemological differences and knowledges from the ground, while DIY bio contributes to the expansion of science to include alternative practices and ways of knowing (see Egert and Allen, 2019). DIYbio practitioners conduct science outside traditional research environments, often in community spaces where members undertake a wide range of activities roughly falling under bioscience: developing low-cost scientific equipment, working on health and environment issues, making bio art, undertaking educational and outreach activities, and building entrepreneurial projects and start-ups (Meyer and Vergnaud, 2020). Partially due to the expansive scope of activities, there are also a range of aims and political goals associated with DIYbio and debates about what counts as DIYbio (Wexler, 2017).

However, existing research has analysed how DIYbio groups, despite their diversity, generally aim to democratise and de-institutionalise science, especially bioscience. Members of DIYbio groups usually work to move science out of the ivory tower of professional research institutions and laboratories and develop alternative models of research and expertise (e.g. Meyer, 2013; McGowan *et al*., 2017; Guerrini *et al*., 2020; Meyer and Vergnaud, 2020). However, while DIYbio generally aims to enable non-professionals without scientific credentials to practice science themselves, DIYbio practitioners often have advanced science degrees and concurrently occupy professional science roles, resulting in significant overlap between DIYbio and institutionalised science (Grushkin *et al*., 2013; Meyer, 2013; Ikemoto, 2017; Guerrini *et al*., 2020). Existing surveys suggest that DIYbio groups disproportionately encompass individuals with advanced degrees; they are disproportionately male and a quarter are students (Grushkin *et al*., 2013; Meyer and Vergnaud, 2020). Their activities are generally, however, guided by disillusionment with mainstream scientific institutions; a desire to open the practice of science to those it impacts; and values like freedom and self-determination to experiment, collaboration and inclusive participation, and open access to scientific knowledge, tools, and protocols (Guerrini *et al*., 2020).

DIYbio can be understood as what Vaage (2016) called ‘fringe science,’ which captures a range of heterogeneous activities at the outskirts of institutionally legitimated science. The fringe notion is a spatial metaphor implying ‘not a firm, distinct boundary or border, but more a blurry, uneven expanse of space’ (Vaage, 2016, p. 113). It can encompass activities from informal local groupings to formal international networks, and from not-for-profit community endeavours to start-ups and alternative technoscientific business models. DIYbio can be seen as a manifestation of fringe science, highlighting its often-marginal nature as forming a liminal sphere of fringe activities. As such, DIYbio may serve, simultaneously, to ‘shape, affirm or move the boundaries of what is considered science, and who is allowed to practice it,’ ‘shifting the current ideas of who is entitled to conduct research in the life sciences, and how such research should be done’ (Vaage, 2016, p. 127).

However, the DIY movement in general, including DIYbio, has also been shaped by overarching socio-economic developments in which citizenship discourses have undergone problematic shifts. Across the world, governments have implemented neoliberal governance models, including public funding cuts, public service privatisation, and broader state rollbacks, accompanied by political discourses encouraging citizens to be active and entrepreneurial, taking responsibility for their own lives rather than relying on state support (Drake, 2011). One effect of neoliberal governance is offloading responsibilities from states to citizens, who are encouraged to cultivate self-governance. As such, DIY has been conjured as an empowering means for citizens to foster greater control over their lives (Drake, 2011). Despite their emancipatory undertones, a consequence of neoliberal shifts has been an appropriation of citizens’ voluntary labour as a low-cost way to facilitate productivity and innovation, where citizens’ DIY mobilisation becomes embedded in the construction of individually responsible, entrepreneurial citizens (Lave, 2012). The displacement of state responsibilities to individuals has included calls for citizen volunteers to take up roles vacated by public service funding cuts (Bloom and Kilgore, 2003). The contemporary DIY movement should be understood in the context of these neoliberal developments, and the celebration of DIYbio as a potentially emancipatory and democratising example of the move towards citizens’ active contribution to science innovation (see e.g. Delgado, 2013; Kuiken, 2016) should also be seen in this light.

While some have celebrated DIYbio, others have raised concerns that it represents a dangerous de-professionalisation or ‘de-skilling’ of bioscience (e.g. Schmidt, 2008; Tucker 2011). For example, some scholars have worried that DIYbio poses threats from bioterrorism to hobbyists with little scientific training accidentally or intentionally releasing harmful organisms into the environment (e.g. Tucker, 2011; Vargo, 2017). Bostrum has even framed it as a key form of contemporary ‘biological aggression’ with the potential to cause societal level harm. ‘DIY biohacking tools might make it easy for anybody with basic training in biology to kill millions,’ including because developments in areas like synthetic biology ‘could produce a discovery that suddenly democratizes mass destruction’ (Bostrom, 2019, p. 455). Vargo has similarly presumed that ‘malicious do-it-yourselves will almost certainly materialise sooner or later’ (Vargo, 2017, p. 69).

DIYbio has also been described as a ‘Wild West’ of science that falls between gaps in existing science oversight mechanisms (Blazeki, 2014). Concerns have been raised over DIYbio practitioners having insufficient (formal) training in safety and ethics and that DIYbio enjoys an unregulated status (Kolodziejczyk, 2017). More specifically, existing science governance frameworks are principally designed to regulate science conducted in institutional contexts, but adapting these frameworks to DIYbio is not always straightforward. For example, institutions mandate ethical review for biomedical research, but DIYbio is generally practiced outside these institutions. Others have argued that these concerns are unfounded, because DIYbio practitioners who are also professional scientists are transporting safety and ethics frameworks from institutional to DIYbio contexts (Sundaram, 2021). DIYbio groups also have a history of collaboration with biosafety specialists and law enforcement (Tocchetti and Aguiton, 2015) and tend to engage in significant safety and ethics self-regulation, often in collaboration with professionals (Seyfried *et al*., 2014; Kuiken, 2016; Guerrini *et al*., 2020).

These concerns are also shaped by conceptions of research ethics. Mainstream approaches to research ethics draw on principle-based ethics, epitomised by Beauchamp and Childress (1979). While principle-based approaches have been the hegemonic way in which biomedical ethics have been framed (Sargent and Smith-Morris, 2006; Finkler, 2008), they struggle to account for the power-endowed ways in which ethical practice is conceived and enacted in different local settings. Individuals and groups understand and practice ethical conduct in diverse ways, shaped by culturally-framed comprehensions within the context of political, economic, and social realities (Finkler, 2008). Principle-based ethics struggle to reliably provide a grounded approach to ethics that would attend to the wider context and local settings where science occurs (Sargent and Smith-Morris, 2006). This includes DIYbio, which is often shaped by alternative conceptualisations of what ethical practice should entail. These do not necessarily align with, and sometimes explicitly challenge, institutionalised ethical conduct norms, as I show below.

The above contestations over the risk of DIYbio can be understood as a part of the dynamics of expectations of new and emerging technoscientific developments. The sociology of expectations literature has documented how new developments are often accompanied with future-oriented expectations that articulate the potential of what science could or should enable (see e.g. Brown *et al*., Webster, 2000; Brown and Michael, 2003; Borup *et al*., 2006; Konrad *et al*., 2017). The expectations are then attached to the new developments as their ‘promise’ or as their peril. Alongside positive promises, fears about potential future risks often run parallel to promissory expectations (Borup *et al*., 2006), exemplified by worries over DIYbio as a ‘Wild West’ of science.

Expectations are not neutral or value-free articulations of what is possible but tend, rather, to express promises and concerns about the future that are framed by valuations of what is desirable or undesirable (Konrad *et al*., 2017). These normative associations have been theorised through the anticipatory concept of ‘visions,’ which ‘often relay a fuller portrait of an alternative world that includes revised social orders, governance structures, and societal values’ and ‘usually imply normative connotations, often being statements of desirable or preferable futures’ (Konrad *et al*., 2017, p. 467). The competing and contested nature of expectations and visions has been highlighted as a key part of visions’ dynamics: the envisioned futures are plural (Brown *et al*., 2000; van Lente and Bakker, 2010).

Understanding expectations and visions of the future is central for understanding technoscientific shifts, especially because expectations are performative. They tend to shape activities in the present, directing them towards the realisation of the future visions that have been manufactured (Brown *et al*., 2000; Borup *et al*., 2006). They are thus not an additional but a constitutive force shaping science and innovation. Sociology of expectations calls for ‘scholarship to engage with the future as an analytical object,’ and examine ‘the forms of action and agency through which the future is both performed (as a temporal representation) and colonized (as a spatial and temporal locus)’ (Brown and Michael, 2003, p. 4). It asks for a shift in the analytical angle ‘from *looking into* the future to *looking at* how the future as a temporal abstraction is constructed and managed, by whom and under what conditions’ (Brown *et al*., 2000, p. 4, original emphasis).

Yet, much of the sociology of expectations has focused on analysing the expectations and visions promoted by professional actors in the technoscientific mainstream, especially academic and industry researchers (and popular media), and often concerning specific emerging technologies or new scientific fields (Hielscher and Kivimaa, 2019). Less attention has been directed towards visions promoted by actors outside the mainstream, including alternative future promises manufactured around the notion of science itself. There is a need to look at how unconventional actors like DIYbio initiatives construct, manage, and perform the future of science, as a socio-technological endeavour, at the fringes of mainstream science. Such an investigation enables scholars to broaden our engagement with the future as an analytical object, shifting focus from the centre to the margins. This is especially pertinent in the present context, in which the boundaries of science are being stretched to accommodate new actors and modes of citizen participation, while citizens are increasingly expected to be active, entrepreneurial, and self-governing.

## Methodology

This paper draws on an analysis of DIYbio initiatives’ websites. Leading individuals (e.g. organisers or founders), often create the website content, meaning that it may not reflect members’ general views. Yet, websites are an important way that groups portray themselves to the world in the digital age. The website analysis focused on DIYbio initiatives’ ‘about us’ descriptions, and mission, value, ethics, safety and security statements. These statements are generally formulated to delineate a collective vision that can be used to communicate central beliefs about a group’s distinctive attributes to people both inside and outside the group, indicate expected behaviour standards, and provide a value-based future direction (Klemm *et al*., 1991).

The website search was conducted in November and December 2019, providing a snapshot of DIYbio initiatives. The DIYbiosphere, an open-source project connecting DIYbio initiatives throughout the world, maintained by the largest international DIYbio network, DIYBio.org (2019), was initially used to identify initiatives. All DIYbiosphere listings were searched for active initiatives, including community laboratories, groups, networks, start-ups and incubators. Start-ups and incubators were included because there is a notable entrepreneurship dimension to DIYbio, highlighting how it incorporates cross-sectoral overflow and hybrid assemblages of professional scientists, community groups, advocacy organisations, and commercial actors. Only initiatives with functioning English language websites were included. Websites with content in more than one language were included if they had enough content in English to ascertain basic information, some ongoing activities, and value-based statements. There is also significant overlap between DIYbio and wider ‘maker’ and ‘hacker’ communities (Davies, 2017). Wider initiatives were included if they had a recognised DIYbio presence.

Eighty-eight initiatives’ websites were included, fourteen of which are (purely) digital networks. The rest are disproportionately based in Anglophone countries, probably reflecting the exclusion of initiatives without English language websites and the disproportionate representation of white, male and urban people in DIYbio (see Grushkin *et al*., 2013; Meyer and Vergnaud, 2020). Thirty-one are based in the USA and twenty-seven in Europe, with six of these in the UK, seven in Germany, five in Canada, two in Australia, and one in Singapore. Only eight are based in Low- and Middle-Income Countries (LMIC) (Brazil, India, Peru, Nepal, Indonesia, Philippines, Nigeria, and Ghana). Most are non-profit community laboratories, but some are start-ups and incubators facilitating DIYbio activities, including investing in and offering comparatively low-cost laboratory facilities for people outside professional science sectors.

The websites were first searched for ‘about us’ content and relevant statements, which were entered into a dataset, alongside information concerning location, name, and URL. Additional information, including rules, policies, and further resources like commentaries and manifestos, were recorded when present. Partnership information and website links were used to identify further initiatives not listed in DIYbiosphere. Additionally, Google keyword searches (‘DIYbio’ ‘and ‘biohacking,’ with ‘laboratory’ ‘lab,’ ‘group,’ or ‘community’) were conducted to identify further initiatives. Keyword searches retrieved between 12 and 24 pages of search results. Building on a strategy developed by others (Turner and Knoepfler, 2016), a minimum of 15 search result pages were reviewed to compensate for limitations such as Google page ranking bias (unless the search retrieved fewer than 15 pages). However, no new relevant initiatives were found this way.

Data were analysed using deductive qualitative thematic analysis (see Braun and Clarke, 2012), to identify and examine patterns in the semantic content and underlying discourses that foreground them. This was used to derive key themes around how DIYbio initiatives conceptualise science and what perspectives and contextual developments underlie these conceptualisations. Two overarching themes emerged across the included initiatives’ self-descriptions: promissory visions and reconfigurations of science promoted by DIYbio initiatives, and DIY oversight relating to DIYbio initiatives’ self-governance and normative visions.

This study was exploratory, meaning that the findings can only be used to understand emerging themes around DIYbio rather than derive conclusive statements. As the analysis was focused on DIYbio initiatives’ self-representations, the findings cannot be used to assess what happens on the ground in DIYbio communities and whether the visions highlighted in online content are translated into practice. Relatedly, based on the website self-representations alone, it cannot necessarily be presumed that all included initiatives constitute collectives because websites claiming to represent collectives might only represent individuals. The findings also have limited contextual, including geographical and cultural, reach, constraining the extent to which they can be presumed to apply beyond the Anglophone, mostly high-income and Western countries where the included initiatives were mostly based. Further, since websites with content in multiple languages were only included if they had sufficient English content, relevant information in other languages was likely missed. Because the search was driven by the DIYbio.org network, some initiatives not recognised by DIYbio.org may have been missed. While the additional online keyword searches aimed to mitigate against this, only a limited numbers of keywords were used.

## Mapping the DIYbio Sphere: Promissory Visions and Reconfigurations of Science

Concordant with existing research (e.g. Grushkin *et al*., 2013; Meyer and Vergnaud, 2020), the included DIYbio initiatives are diverse, ranging from large, well-organised international networks to funded community laboratories with academic and corporate connections to small-scale informal local groupings. They use various labels, sometimes concurrently, to describe themselves, including DIYbio, biohacking, and citizen science, but also biomarker, biotinkering, biopunk, bioinquisitive, and hackstronaut. Their workspaces are variously called a community lab, open biolab, biostudio, underground science space, counterculture lab, and hive, among others. This heterogeneity was significant enough to mean the initiatives cannot be easily grouped under discernible activity types, but there are notable patterns.

The initiatives most prevalently fall under conceptual frameworks aligning with professional science, and explicitly include professional scientists among their founding or core membership. For example, many have formalised educational and public engagement directives like training workshops delivered by professionals and mentoring schemes to involve people in science. An example is Genspace in New York, which is among the older and formally organised DIYbio labs with well-established links to professional science institutions. Genspace (2019) represents itself as ‘a nonprofit organization dedicated to promoting science literacy through citizen access to biotechnology,’ through ‘STEM educational outreach, cultural events, and a platform for science innovation at the grassroots level.’ The organisation believes that ‘the best way to inform twenty-first century dialogue about science is to have stakeholders understand it from a hands-on perspective’ (Genspace, 2019). It runs educational activities ranging from bioinformatics to plant DNA barcoding to social implications of bioscience, accessible to people without science backgrounds, and delivered by instructors ‘from top institutions around New York City’ (Genspace, 2019).

Similar initiatives include the Melbourne-based BioQuisitive, a well-established community laboratory that hosts educational events and workshops. BioQuisitive’s (2019) website states that community laboratories provide ‘a great connection between the general public and institutions of science,’ a chance for ‘academics and industry professionals’ to ‘engage a wider audience on their work,’ and opportunities for ‘students and members of the general public to learn and practice hands on skills.’ Other initiatives, like the Open Wet Lab (OWL) in Trento, Italy, are associated with universities and have a membership of mostly bioscience students and alumni.

These initiatives, also including, for example, MIT DIYbio in Cambridge, Massachusetts, Biomakespace, in Cambridge, UK, and HiveBio lab in Seattle, foster links and collaborations with universities and employ institutionally legitimated terminology like ‘educational outreach’ and ‘public awareness of science.’ They envision a future of democratised access to science and correspondingly aim to facilitate entry for non-professionals. Their visions for what science does and should entail, and the access they provide to science is, however, largely consistent with institutionalised notions of participatory public engagement. Further, they operate by following standard scientific methods. Their overlap with professional and student groups is also suggestive of the kinds of practical learning they promote: training in institutionally validated scientific procedures.

The extent to which different initiatives share the above kinds of characteristics varies in degree. For example, some have more established connections to institutional science and more formal training and outreach programmes than others, and there are different conceptualisations of what democratised access to science should entail in practice. While they challenge who can do science and where, their aims and visions neither necessarily undermine institutionally legitimated conceptualisations of what science should entail, nor the epistemic authority of professional scientists. Rather, they can supplement them by framing DIYbio as belonging within legitimate science engagement activity.

While few DIYbio initiatives based in LMICs were included in the study, all but one of the LMIC organisations advances promissory visions that were somewhat differently framed than those previously described. Apart from the university-affiliated (Art)ScienceBLR public laboratory in Bengaluru, India, initiatives in LMICs, including start-ups and incubators, articulate visions of science directed towards destabilising global power and resource disparities. For example, the incubator Kumasi Hive (2019) in Ghana aims to ‘lowering the barriers to creating local small-scale manufacturing businesses for products needed in the community.’ Their sponsored initiatives include the Hive Biolab (2019) community laboratory (also called Open Bioeconomy lab), which hopes to ‘contribute to creating an open, sustainable and equitable bioeconomy’ in Ghana and across the Global South. The Hive Biolab develops open-source toolkits to locally manufacture enzymes and open approaches to intellectual property. It aims to overcome restrictions concerning ‘who has the ability to perform biological research and agency to shape the direction of biotechnology’ – restrictions including ‘proprietary models of ownership’ and ‘lack of access to knowledge and research tools’ especially in low-resource settings (Hive Biolab, 2019). Similarly, Conector Ciencia (2019), a DIYbio network that runs events across Brazil, emphasises that ‘in the Brazilian context … we face challenges such as lack of resources, high social and education inequalities and large-scale environmental challenges.’ Conector Ciencia (2019) promotes ‘the culture of DIYBio as an innovative social movement that leads to more diversity in the making of science and technologies’ but aims to ‘apply this new way of doing science’ in ways that ‘contextualize practices within Brazilian pressing issues.’

These initiatives advance visions around democratised access to science, educating publics, and facilitating non-professionals’ entry into science in ways similar to High-Income Country (HIC) initiatives like Genspace and BioQuisitive. Yet, their key aims centre local community needs – contextualised by socio-economic disadvantage in relation to global inequities in bioscience – as drivers of DIYbio. Initiatives like Kumasi Hive, Hive Biolab, and Connector Ciencia, but also Biomakers lab in Peru, for example, manufacture promissory visions geared towards addressing these inequities, including by fostering not just the development of individual tools but broader bioeconomies.

In the context of global economic resource and technoscientific capacity disparities, HICs principally drive the professional and commercial development of new bioscience. Commercially developed biotechnology is often patented and priced in ways inaccessible for low-resource populations, and the direction and distribution of research investment are not properly aligned with global demands. At the same time, regions with the highest disease burdens also have the lowest proportion of biomedical workers (WHO, 2018). DIYbio initiatives in LMICs promote the subversion of these inequities by facilitating local community-driven open-source bioscience, enabling communities to create their own knowledge and tools and directly meet local needs. They promote access to science as a facilitator of socio-economic change but in ways that reject the global power hierarchies that currently structure bioscience. Their conceptualisations and future visions of science broadly align with conventional delineations of science and scientific methods, but they explicitly politicise bioscience with respect to global power relations. They call for local people to ‘do science themselves’ and envision a ‘new way of doing science’ in contextually relevant ways that challenge who should make decisions about research priorities and where.

Several DIYbio initiatives in HICs express more radical visions that re-configure what science is or could become. One of these is the New York-based Hackstronauts initiative, whose website focuses on loosely on biotechnological and pharmacological self-enhancement, including cognitive enhancers or ‘nootropics,’ wearable technologies, and pharmacologically induced ‘altered states.’ Hackstronauts (2019) describe themselves as

inventors, explorers, daredevils and yes, human guinea pigs. We are true believers and virulent skeptics. We are the crazy people that throw ourselves off of moving band-wagons. … Some will say we’re cheats, line-cutters and outlaws but we just like to trash rule books. The world is already so full of ‘should’ we’re going to fill it with ‘could’. … We have a vision of a planet infected by curiosity and we want it NOW.

While Hackstronauts (2019) express a commitment ‘to the scientific method in both its classical practice and abstract principles,’ they conceptualise scientific ‘observation’ in terms of ‘superhuman performance,’ and ‘question’ phenomena in terms of whether they can be ‘hacked,’ for example.

Alternative initiatives in Spain espouse differently framed political visions: the Pechblenda lab and GynePunk collective in Calafou, and Quimera Rosa, initially based in Barcelona but now identifying as a nomad organisation. Pechblenda describes itself as a ‘non-patriarchal TransHackFeminist space where free knowledge springs from raw experimentation … and self-education’ (Pechblenda, 2019). Pechblenda is part of a larger anti-capitalist community in Calafou, and its members are explicitly influenced by wider techno-feminist and trans-feminist politics, including Donna Haraway (1991) and Paul Preciado (2013). They describe themselves as ‘geek whores’ and ‘cyborg witches’ who ‘want to hack and recodify everything that is static and programmed by social and technological imposition.’ PECHBLENDA is injected into our veins as an antidote to the heteropatriarchal arrogance that surrounds us’ (Pechblenda, 2019). Similarly, the GynePunk collective, affiliated with Pechblenda, explicitly positions itself in opposition to conventional biomedicine, including gynaecology and gender-affirming care. GynePunk states that they ‘don’t want to be forced to enter into their hygienist temples, in veiled body jails, in those fabrics of corporal standardization,’ but rather aim to reclaim (especially transgender) women’s bodies from ‘compulsive dependency of the fossil structures of the hegemonic health system machine’ (Hackteria, 2015). GynePunk provides open-source protocols and knowledge that people can use to perform their own basic sexual and reproductive healthcare. They combine biomedicine with ‘knowledge that comes through the experience of each body,’ ancestral body wisdom, healing rituals, and art (Hackteria, 2015), stretching the boundaries of knowledge about the (sexual and reproductive) body beyond conventionally legitimate(d) science.

These initiatives’ visions re-configure not only who can or should practice science but also what science means. They overtly frame their practices as alternative to conventional biomedicine and bioscience, and especially Pechblenda and GynePunk, conceptualise both institutionally legitimated science and their alternative modes of science as explicitly politicised endeavours. GynePunk positions mainstream biomedicine as oppressive and medically normalising, while framing their alternative conceptualisation of sexual and reproductive health as emancipatory and liberating. By describing the ‘hegemonic health system’ as ‘fossil,’ they also frame it as anachronistic in ways enabling them to simultaneously position the kinds of biomedical practice they promote as temporally in the present and future. This perspective re-frames epistemic authority as arising from embodied experience and alterative knowledge practices as well as from conventional science. They claim a radically new kind of science by re-framing the aims and epistemic basis of science by constructing promissory visions that are purposefully political, counter-hegemonic and future-oriented. Their visions centre around re-fashioning of the world by re-purposing the tools of science and using them to improve oneself, in one’s own terms, or to advance alternative political goals.

Despite their diverse goals and commitments, the purpose statements that DIYbio initiatives put forward are centrally promissory and driven by visions of desired futures. While what these futures look like varies, the promissory visions are performative in that they shape how DIYbio groups practice and conceptualise science. Whether they advocate democratised access to science through participatory public involvement, aim to address local needs and global disparities around bioscience, or imagine radically alternative possibilities where the epistemic bases of science are unmoored from ‘heteropatriarchal arrogance,’ DIYbio initiatives re-define what doing science can mean, who can do it, and where. However, as Meyer (2013) observed, most DIYbio initiatives have links to professional science, both through their membership that includes professional scientists and their collaborations with professional science institutions. This means that DIYbio is still, indeed, largely ‘a “promised” amateur science’ and ‘not yet an already established “amateur science”’ (Meyer, 2013, p. 123).

DIYbio activities and the visions that shape them also take place within wider contexts where neoliberal discourses are shaping the landscape of science innovation including promoting self-responsible entrepreneurial citizenship. The influence of these discourses is visible in some DIYbio initiatives’ statements of purpose, especially incubator companies: for example, a key aim of the Kumasi Hive (2019) incubator is ‘promoting entrepreneurship’ in the local community to facilitate innovation that can be commercialised. While Pollio (2019), among others, has noted that technology incubator companies’ operational motivations can incorporate multiple rationalities, especially in places of socio-economic marginality, they are often driven by commercial interests, stretching market forces to new territories and producing entrepreneurial subjects that can foster new markets. Some grassroots community laboratories explicitly promote the idea of DIYbio as entrepreneurial citizen innovation. Counter Culture Labs (2019) in California, for example, likens the DIYbio movement to the high-technology corporate innovation hub, Silicon Valley, in San Francisco:

Silicon valley [*sic*] was born out of garage workshops and hobby clubs, the precursor to today’s hacker spaces. We believe that if we build a place with the tools and the community to support them, tomorrow’s great innovators in biotechnology can be born out of spaces like Counter Culture Labs. Biology is the technology of the 21st century.

The overlap between DIYbio community laboratories and start-ups highlights that while DIYbio may challenge dominant power relations around science, it can simultaneously support neoliberal governance that appropriates citizens’ entrepreneurial but unpaid labour to facilitate innovation (see also Delfanti, 2013; McGowan *et al*., 2017).

## DIY Oversight: Self-governance and Normative Visions

DIYbio initiatives’ promissory visions also give rise to ideas about science governance and normative commitments, shaping how ethics and safety are conceptualised. Many DIYbio initiatives have formalised ethical codes, building on conventional principle-based approaches to bioethics and biosafety guides, some of which were developed in collaboration with professionals, drawing from institutionalised ethics and safety requirements. While some individual DIYbio initiatives have their own principle-based ethics codes, DIYbio.org (2019) developed the most influential codes (the global DIYbio network encompassing the DIYbio initiatives included in this study). Many groups list or link to the DIYbio.org codes on their websites. These codes consist of two overlapping but slightly different ethics codes for North American and European DIYbio initiatives, originating from a series of congresses organised by DIYbio.org in 2011 that assembled members of local DIYbio groups from North America and Europe. While the group intended to develop a single global code, two separate regional codes were developed. The European code was formulated during a congress in London attended by DIYbio groups’ representatives from England, France, Germany, Denmark and Ireland. A month later, representatives from California, New York, Texas, and Massachusetts developed the North American code in San Francisco (DIYbio.org, 2019). The codes share principles of transparency, open access, safety, peaceful purposes, and education, but while the North American code lists open access as the first, and presumably most important, principle, transparency is the European code’s first principle. The European code also incorporates modesty, respect, responsibility, accountability, and community, while the North American code includes tinkering and the environment (i.e. environmental protection). Additionally, DIYbio.org (2019) has partnered with bioethics and biosafety professionals, including from the Woodrow Wilson Centre, to develop ethics and safety standards and establish a biosafety advisory board and an ‘ask a biosafety expert’ forum where certified professionals answer biosafety questions.

The development of codified ethics and biosafety standards reflects institutional scientific research governance models and likely seeks to mitigate concerns over DIYbio as a ‘Wild West’ of science. By collaborating with professional bioethicists and biosafety experts, DIYbio.org can claim credibility for their activities as ethical and safe because of association with institutionally legitimated expertise. Being principle-based, DIYbio.org’s ethics codes also reflect mainstream approaches to research ethics applied in institutional contexts. However, their principles are notably different from mainstream bioethics principles, which emphasise autonomy, beneficence, non-maleficence, and justice, following Beauchamp and Childress (1979). They promote alternative ethical priorities especially through the commitments to open access and education. Nonetheless, they are, like principle-based bioethics generally, built on the idea that there exists some sense of common morality that can be codified and applied cross-contextually (Sargent and Smith-Morris, 2006). Indeed, DIYbio.org (2019) notes that ‘one motivation for establishing DIYbio.org … is to help create frameworks for best practices worldwide.’ Yet, the challenges with this are illustrated by the continued existence of two regionally divided DIYbio.org ethics codes.

As Eggleson (2014) observed, DIYbio.org likely retains two separate regional ethics codes years after their drafting because of cross-Atlantic cultural differences. The North American code’s lesser emphasis on relational/communal values like community but also respect, responsibility and accountability is likely connected with the individual-centred cultural values prominent in the USA, including self-sufficiency, autonomy and individual rights. In contrast, the welfare state models of most European countries centre collective responsibilities and resource sharing. The codes’ divergence is also, however, likely connected with differences in the political and governance context in which European and North American DIYbio groups operate. In particular, the European Union’s science governance context has been shaped by the responsible research and innovation (RRI) agenda^1^ institutionalised within European science programmes (see Simone, 2018). The roots of RRI in Europe can be traced to public and policy debates around research integrity and the social, legal and ethical implications of science in controversial areas like synthetic biology, which sparked efforts to make research and innovation more transparent, accountable, and responsive to citizens, including via public involvement (Owen *et al*., 2012). The European DIYbio.org ethics code’s emphasis on transparency and incorporation of modesty, respect, responsibility, and accountability can be seen as embedded within the broader European science governance agendas where RRI has become central and as a way to foster credibility for DIYbio by aligning ethics with institutionalised science governance priorities.

Some DIYbio initiatives, however, advance alternative visions of science ethics and governance that challenge institutionalised frameworks. A prominent example is the *Biopunk Manifesto*, initially delivered in Meredith Patterson’s talk at a 2010 conference on ‘outlaw biology’ (see Kelty, 2010). The *Biopunk Manifesto* explicitly builds on a *Cypherpunk’s Manifesto* written by Hughes (1993), the founding member of the cypherpunk movement, which called for anti-government resistance to regulation and information control in cyberspace. Indeed, it has been argued that DIYbio more generally represents a transfer of the ethics of earlier generations of computer hackers (emphasising decentralisation, resistance to authority, free access to technology and information, and formal equality of those who access it, see Levy, 2001) into the life sciences (Delfanti, 2013). Drawing from but re-framing these ethics in DIYbio terms, Patterson’s (2010) manifesto rejected the idea that ‘science is only done in million-dollar university, government, or corporate labs’ and asserted the ‘right of freedom of inquiry, to do research and pursue understanding under one’s own direction.’ ‘Biopunks,’ she stated, ‘take responsibility for their research’ and ‘are acutely aware that our research has the potential to affect those around us,’ but they also ‘reject outright the admonishments of the precautionary principle, which is nothing more than a paternalistic attempt to silence researchers by inspiring fear of the unknown’ (Patterson, 2010). The ‘punk’ notion in both the cypherpunk and biopunk manifestos draws from the punk music scene, characterised by an anti-establishment stance and DIY ethos (Dunn, 2008).

Mirroring or directly building on these normative commitments, many DIYbio initiatives frame ethics not in terms of formalised codes or principles but of bottom-up oversight and self-governance. Often, these frames do not directly distinguish among ethics, respectful conduct, safety, or security norms but rather advocate what can be understood as a normatively framed attitude towards science. Indeed, while a quarter of the initiatives included in this study have formal ethics or safety codes on their websites, others mention ethics only via short statements left open to multiple interpretations. The SoundBio lab (2019) in Washington, for example, simply states that members ‘should err on the side of ethical caution,’ ‘follow the principle of “do no harm”’ and ‘“do good” in our community and world, recognizing the complexity of the concept of “good”’ (SoundBio, 2019).

Initiatives like Pechblenda and GynePunk promote normative commitments expressly exceeding institutionalised biomedical ethics, in ways explicitly embedded within their broader anti-institutional, counter-cultural feminist visions. Ethical commitments are articulated, to quote GynePunk, in opposition to the ‘moral perversion’ of medical institutions, including ‘bureaucratic, statistical forms that performs a role of popular judges of your practices, capacities or choices … seeking data about your promiscuity, drug use, sexual orientation, hygienic practices, or squat relations’ (Hackteria, 2015). Such articulations of what is and is not ‘moral’ frame normative directions in terms of wider politics and moral commitments that should be achieved (i.e. non-patriarchal, feminist and emancipatory science), rather than in terms of particular principles or codes that should be followed to comply with ethical practice norms.

More moderate initiatives used the idea of bottom-up community oversight and self-governance, including the London Hackspace, which includes the London Biohackspace DIYbio group. The London Hackspace (2019) does have some (relatively loosely defined) conduct rules, but these are accompanied by the statement:

as hackers we hate making rules almost as much as we hate following them, so we really want to keep the number of rules to a minimum. We can only do this if members and visitors observe the spirit – not just the letter – of these rules.

The London Hackspace rules, encompassing general ethical, safe, and respectful conduct, are framed in ways that make both the enforcement and interpretation of concepts like safety the members’ responsibility. For example, they include rules like ‘don’t use tools unless you’re sure you know how to do so safely’ and ‘if you see someone working in an unsafe way, it’s your duty to stop them and let them know’ (London Hackspace, 2019). Similarly, while Biocurious (2019) in California highlights that they do require members to operate safely and follow basic safety guidance, they do ‘not exercise editorial control over the science done in the lab.’ Further, they ‘do not make judgement calls on whether an experiment is good science or not, or whether it is ethical’ but rather expect members to make their own ‘responsible decisions.’ This represents a form of self-governance in which what constitutes safe or ethical practice, and the responsibility to ensure it, is deferred to members, who are expected to govern themselves without rigorous top-down rules.

These conceptualisations provide an alternative to institutionalised science governance frameworks and arise out of DIYbio initiatives’ more expansive promissory visions around science, shaping how they frame ethical and safe practices. Where the aim is to re-configure the practice of science by fostering science as a democratised, open, and accessible endeavour, ethics and safety also become bottom-up, self-directed issues. This is most apparent for initiatives advancing explicitly transformative science futures but is also present when leaders emphasise the spirit rather than the letter of rules. DIYbio initiatives are thus manufacturing alternative futures of do-it-yourself governance as well as science itself. That DIYbio initiatives are generally not encompassed within institutional research governance frameworks enables them to re-frame what ethical and safe conduct mean. This can be seen as a form of resistance to ethics from above, including subversion of the institutionally legitimised expertise of bioethicists as well as scientists. Even the ethical commitments expressed in more formalised DIYbio ethics codes like the DIYbio.org codes enact reformulated ethics that move, albeit less radically, towards bottom-up oversight. Many of the DIYbio.org ethical principles align more closely with counter-hegemonic aspirations than institutionalised ethics. Open access, in particular, is described as a normative duty to promote ‘decentralized access to biotechnology’ (DIYbio.org, 2019).

It is notable that most DIYbio initiatives advocate, in different ways and different degrees, self-governance, decentralisation and deferral of ethical and safe practice to their members. This focus on self-governance should be considered in the context of neoliberal political discourses and governance: especially ideas expressed in the *Biopunk* manifesto that are reflected across many DIYbio initiatives’ ethics and safety statements and express libertarian conceptualisations of freedom and anti-government politics that support rollbacks of state authority, including over science. The promotion of self-responsible DIYbio practitioners, who should govern themselves without relying on institutions to govern for them, as a part of the promise of DIYbio lends support to neoliberal discourses that frame the offloading of state responsibilities onto citizens as an empowering means of giving citizens greater agency and control.

## Conclusion

This paper used DIY biology (DIYbio) as a lens to explore the changing meaning of science in the current context, in which people can practice science – including bioscience – outside institutional science in unprecedented ways. Furthermore, it investigated how such practices promote discourses of neoliberal citizenship. Drawing on thematic analysis of DIYbio initiatives’ websites, it focused on DIYbio initiatives’ promissory visions, asking how science is conceptualised and what political and normative visions and contextual factors underlie these conceptualisations.

The sociology of expectations literature has documented how new technoscientific developments reflect future-oriented expectations that articulate the future ‘promise’ of science. These expectations have been theorised through the anticipatory concept of ‘visions,’ which incorporate valuations of the kinds of futures various actors see as desirable (or undesirable). These manufactured visions tend to be contested and performative. The envisioned and expected are pluralistic, and they also shape the direction of technoscientific activities in the present. Yet, much of the existing literature has focused on analysing the expectations and visions promoted by professional actors in the technoscientific mainstream. This paper builds on and extends these areas of the sociology of expectations by showing how alterative anticipatory visions of desirable futures are manufactured and performatively enacted by unconventional actors at the fringes of mainstream science.

While highly diverse, DIYbio initiatives advance promissory visions expressing desired futures of what science could become, which shape the activities participants undertake, and foreground how science is delineated and stretched to encompass new domains. Most initiatives envision science as a democratised endeavour and aim to widen participation but in ways supporting institutionalised definitions of (participatory) public involvement in science, fostered through links with institutionalised science. In Low- and Middle-Income Countries, this is supplemented with visions to dismantle global inequities around bioscience and enable local communities in under-resourced settings to do science themselves to address local priorities. Some High-Income Country DIYbio initiatives also envision radically alternative futures, advancing conceptualisations and practices that re-erect the epistemic bases of their activities beyond institutionally legitimated scientific procedures to advance political goals challenging conventional power hierarchies. Despite their diversity, DIYbio initiatives’ promissory visions offer an alternative to mainstream science, providing a different basis and sometimes alternate foundation for science and stretching its boundaries to include unconventional actors, practices, and knowledges.

These movements also shape how the governance of science, including ethics and safety, is conceptualised. Some DIYbio initiatives, most notably the DIYbio.org network, have developed codified ethics and safety standards that partially reflect institutionalised research governance frameworks, including collaborations with bioethics and biosafety experts, lending credibility for viewing their activities as ethical and safe. Others promote alternative models of self-governance and bottom-up oversight that re-configure governance as a DIY endeavour, including in terms of normative commitments. Even codified DIYbio ethics express commitments to bottom-up oversight. This is part of the construction of promissory futures that re-configure the normative basis of science, framing governance as a bottom-up, community-driven effort that moves beyond and destabilises not only mainstream governance frameworks but also the meaning of what ethical and safe science entails.

All DIYbio initiatives included in this study represent science as political and normatively driven. They intentionally blur professional versus community spaces, experts versus amateurs, and scientists versus citizens in ways that offer visions different from the currently hegemonic conceptualisations of what science is or should entail. It is because of their fringe position in relation to institutionally legitimated science that DIYbio initiatives can blur these boundaries and advance alternative visions, and in so doing, re-imagine what science could be. Different DIYbio initiatives have different relationships with and ideas about the role of mainstream conceptualisations of science within DIYbio. While some align themselves with institutional science and credentialed experts, others explicitly reject them and re-frame epistemic authority as arising from elsewhere, such as embodied experience and alterative knowledge practices. DIYbio can thus affirm institutionally legitimated notions of science or shift or even radically undermine them. Yet, they all actively re-envision science, promoting futures with new, unconventional science practitioners, heterogenous activities, and alternative normative directions.

Looking at how the fringes of mainstream science envisioned and performatively enacted the future of science enriches the existing sociology of expectations scholarship, which has focused on the technoscientific mainstream. In particular, it expands theorisation of the anticipatory concept of ‘visions,’ and the role of visions in technoscientific developments in ways that are fit to address wider socio-scientific and governance shifts in the present context. DIYbio initiatives’ promissory visions performatively shape the emerging DIYbio sphere, but these visions also shed light on the context in which science is changing to accommodate more active and participatory roles for publics and citizens. Systemic shifts towards multilateral public engagement approaches and the promotion of participatory or DIY citizenship are opening space for new and unconventional re-envisionings of science performed outside the mainstream. While the wider implications of these re-envisionings remain to be seen, they highlight how the futures of science are constructed and contested not only at the centre of scientific innovation but also at the margins by fringe actors who are imagining and performing science in alternative ways. These alternatives exemplify how the meaning of science and who can practice it are currently being re-configured by both conventional and unconventional actors, and they highlight the central role of promissory visions in shaping these re-configurations. In so doing, they also show the importance of broader engagement with the future as an analytical object at the fringes of science.

It is also important to direct attention to the wider socio-economic context that is shaping the construction and manifestations of DIYbio initiatives’ promissory visions, and the configurations of science this context promotes. When DIYbio groups frame science as a DIY activity and promote futures where citizens take science into their own hands and govern themselves, they lend support to neoliberal ideals of the entrepreneurial citizen who takes responsibility for their life rather than relying on public infrastructures and government systems to provide and govern for them. This neoliberal framework includes the promotion of citizens’ un-paid labour to facilitate innovation, but the extent to which one can occupy the entrepreneurial citizen position, including the ability to engage in DIYbio, is unevenly distributed due to existing structural inequalities. Indeed, promissory visions of science as a DIY activity are advanced in ways that harness the entrepreneurial, self-governing citizen subject position as part of the future promise of DIYbio. Taking on this subject position relies on individuals being willing and able (including having the requisite spare time and funds) to perform unpaid scientific labour. That DIYbio practitioners today are disproportionately highly educated urban men highlights how the ability to occupy the position of DIYbio practitioner is socially and politically conditioned by questions around who shares, and can engage in, the promise of DIYbio.

## Note

1 My use of RRI is not an intervention into the theoretical literature around the concept but only highlights how the wider regulatory context shapes DIYbio in Europe.
